# Child with a Tail

**Published:** 2013-10-26

**Authors:** Naeem Liaqat, Asif Iqbal Sandhu, Feeroz Alam Khan, Ejaz Ehmed, Sajid Hameed Dar

**Affiliations:** Department of Pediatric Surgery, Services Institute of Medical Sciences, Lahore, Pakistan

**Keywords:** Spina bifida, Spinal dysraphism, Congenital tail, Tethered spinal cord

## Abstract

Spina Bifida occulta usually presents with some cutaneous stigmata e.g. hair patch, sinus, lipoma, hyperpigmented skin and very rarely a congenital tail. A congenital tail may and may not be associated with spina bifida occulta and tethered cord. A four month old male child presented with congenital tail which was associated with spinal dysraphism and caused tethering of the cord itself. The tail and tethering lesion were excised successfully.

## INTRODUCTION

Spina bifida occulta may rarely present with a congenital tail but in such cases, mostly the tethering lesion is other than the tail. We present a case of four month old male child with a tail like structure which was associated with spina bifida occulta.

## CASE REPORT

A four month old male child presented with a tail like structure on the lower back since birth. There were no symptoms of tethered cord. On examination, one cm long tail like structure, attached to the sacro-coccygeal region in the midline just above natal cleft noted [Fig 1]. The structure had no voluntary movements. It was soft in consistency. Buttocks and natal cleft were normal. There were no other associated congenital anomalies present. Patient was investigated for spina bifida occulta and MRI was done. The lesion was of soft tissue intensity having no communication with spinal cord [Fig 2]. Surgery was planned for cosmetic purposes. After making an incision and dissection, a fibrous cord was found extending underneath the tail, communicating with filum terminale [Fig 3]. Tethering was released, dura and lumbosacral fascia were closed after excision of tail. Histopathology report showed it to be composed of skin and adipose tissue.

**Figure:**
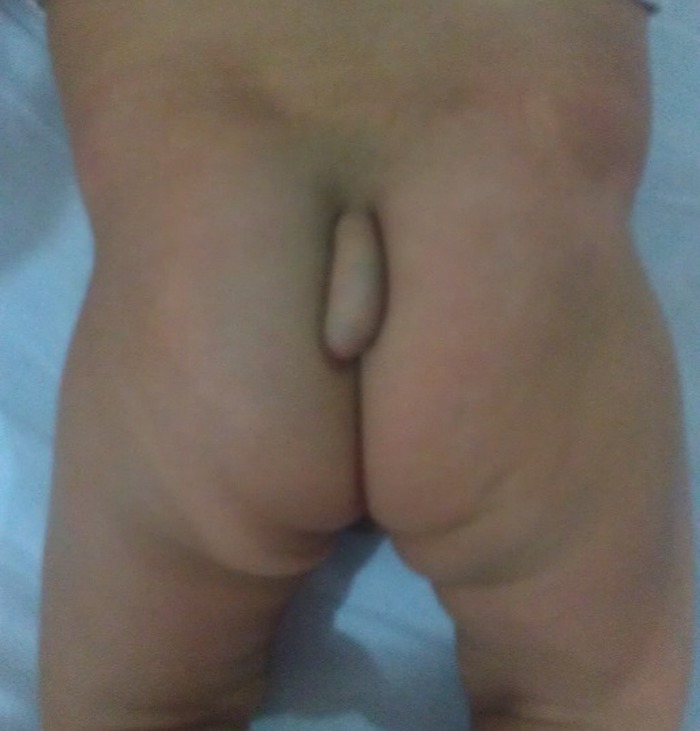
Figure 1:Child with a tail

**Figure:**
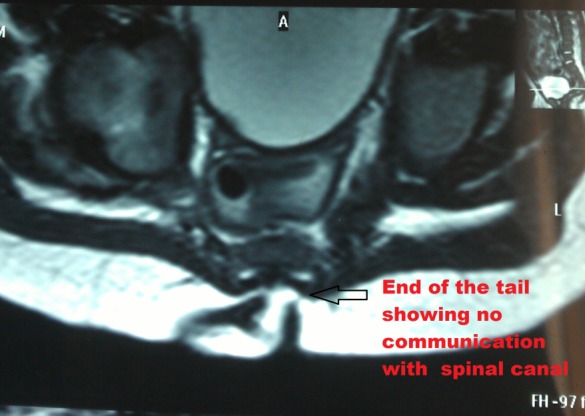
Figure 2:MRI showing no communication with spinal cord.

**Figure:**
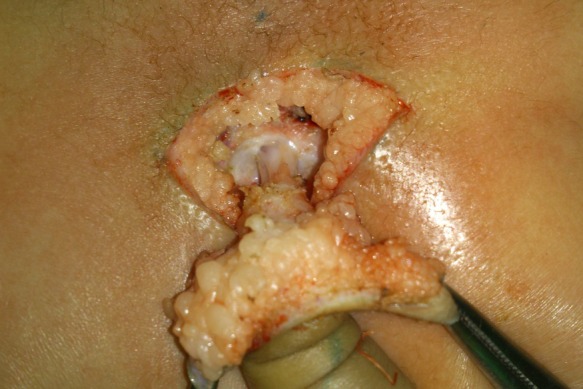
Figure 3:Tethering band is visible.

## DISCUSSION

The incidence of cutaneous lesions along the cranio-spinal axis in general population is about 3%; the cutaneous signs are present in 50% of the patients with spina bifida occulta.[1] Frank et al reviewed 59 cases of human tail and noted incidence of spinal dysraphism in 49% of cases and tethered cord in 20% of patients.[2] The pathology behind formation of tail is thought to be focal premature dysfunction of the neural tube. The unfused neural tube exposes the paraxial mesoderm to the dorsal aspect of the neural ectoderm and induces formation of fatty elements and lipoma. This fatty element formation prevents neural tube fusion and formation of attachment of fatty elements with neural structures, leading to tethering of cord.[3] Congenital tails have been classified into true tail and pseudotail. True tails contain all type of soft tissues like muscles, vessels and adipose tissue while pseudotails have bone, cartilage and remnants of notochord. Another practical classification is division on the basis of association with spinal dysraphism.

All cases of congenital tail need to be fully investigated because of high association with spinal dysraphism.[4,5] Rarely the tail might lead to tethering itself through a fibrous band as found in our case.[6] MRI is usually helpful but surgeon should be very meticulous during excision of tail so as to identify any tethering of spinal cord.

## Footnotes

**Source of Support:** Nil

**Conflict of Interest:** None declared

